# The Involvement of the Oxidative Stress Status in Cancer Pathology: A Double View on the Role of the Antioxidants

**DOI:** 10.1155/2021/9965916

**Published:** 2021-08-05

**Authors:** Kamal Fatima Zahra, Radu Lefter, Ahmad Ali, Ech-Chahad Abdellah, Constantin Trus, Alin Ciobica, Daniel Timofte

**Affiliations:** ^1^Faculty of Sciences and Techniques, Laboratory of Physical Chemistry of Processes and Materials/Agri-Food and Health, Hassan First University, B.P. 539, 26000 Settat, Morocco; ^2^Center of Biomedical Research, Romanian Academy, 8th Carol I Avenue, 700506 Iasi, Romania; ^3^Department of Life Sciences, University of Mumbai, Vidyanagari, Santacruz (East), Mumbai 400098, India; ^4^Faculty of Sciences and Techniques, Laboratory of Physical Chemistry of Processes and Materials, Hassan First University, B.P. 539, 26000 Settat, Morocco; ^5^Department of Morphological and Functional Sciences, Faculty of Medicine, Dunarea de Jos University, 800008 Galati, Romania; ^6^Department of Biology, Faculty of Biology, Alexandru Ioan Cuza University, 11th Carol I Avenue, 700506 Iasi, Romania; ^7^Faculty of Medicine, “Grigore T. Popa”, University of Medicine and Pharmacy, Strada Universitatii 16, 700115 Iasi, Romania

## Abstract

Oxygen-free radicals, reactive oxygen species (ROS) or reactive nitrogen species (RNS), are known by their “double-sided” nature in biological systems. The beneficial effects of ROS involve physiological roles as weapons in the arsenal of the immune system (destroying bacteria within phagocytic cells) and role in programmed cell death (apoptosis). On the other hand, the redox imbalance in favor of the prooxidants results in an overproduction of the ROS/RNS leading to oxidative stress. This imbalance can, therefore, be related to oncogenic stimulation. High levels of ROS disrupt cellular processes by nonspecifically attacking proteins, lipids, and DNA. It appears that DNA damage is the key player in cancer initiation and the formation of 8-OH-G, a potential biomarker for carcinogenesis. The harmful effect of ROS is neutralized by an antioxidant protection treatment as they convert ROS into less reactive species. However, contradictory epidemiological results show that supplementation above physiological doses recommended for antioxidants and taken over a long period can lead to harmful effects and even increase the risk of cancer. Thus, we are describing here some of the latest updates on the involvement of oxidative stress in cancer pathology and a double view on the role of the antioxidants in this context and how this could be relevant in the management and pathology of cancer.

## 1. Introduction

*In vivo* synthesis of reactive oxygen and nitrogen species highlights either a physiological role necessary for normal cell function or oxidative stress characterized by excessive ROS production, thereby altering and damaging intracellular biomolecules, including nucleic acids, proteins, and lipids [1, 2]. Oxidative damage has been described as a serious mechanism in the initiation and progression of cancer.

Cancer is the most worrisome health problem that has received worldwide attention in the past decades. Currently, it is the second leading cause of death in developing countries after cardiovascular mortality [3]. More than 14 million new cancer cases occurred worldwide in 2012, according to the International Agency for Research on Cancer (IARC). The number of cancer deaths increased by 1.5 million, from 6.7 in 2002 to 8.2 million in 2012 [4]. By 2030, the global burden is expected to reach 21.7 million cancer cases and 13 million cancer deaths [5]. This estimate of future burden growth is attributable to physical and environmental risk factors highlighted by globalization and socioeconomic metamorphoses such as pollution, UV radiation, adoption of new lifestyle, and unhealthy diet (poor diet, physical inactivity, smoking, etc.) [6].

There are many factors behind the process of carcinogenesis. Oxidation of DNA, proteins, and lipid peroxidation reactions generated by reactive intermediates, produced by oxidative stress, plays a major role in carcinogenesis. It has been suggested that 8-hydroxy-2-deoxyguanosine, malondialdehyde (MDA), 4-hydroxy-2-nonenal (4-HNE), and carbonylated proteins, by-products of oxidative damage, have mutagenic potential. Increased plasma and tissue concentrations of these oxidative stress second messengers have been reported in lung, gastric, colon, and breast cancers [7–10]. These findings call attention to the role of antioxidant deficiency or increased free radicals in producing such effects and provide a starting point for research into the therapeutic potential of antioxidants in cancer, which was later confirmed to be related to cellular and genetic damage induced by reactive forms of oxygen [11].

The growing knowledge of the mechanisms of oncogenesis related to oxidative stress has allowed the development of several treatments largely represented by the class of complementary alternative therapies, to target a specific mechanism without affecting quality of life, in contrast to less selective conventional chemotherapies [12]. In the last decades, antioxidant molecules have been recognized as one of the most effective forms of alternative and complementary therapy integrating a double therapeutic and preventive facet [13]. They are classified into different groups according to their properties: endogenous enzymatic antioxidants, endogenous nonenzymatic antioxidants, and exogenous antioxidants.

Thus, it was long considered antioxidants to be defensive weapons that can help prevent and suppress the development of cancerous processes, but lately a body of scientific evidence is emerging to challenge the efficacy or even the safety of these complementary alternative treatments calling for a reconsideration of a new paradoxical concept of double-edged antioxidants [14]. The topic on where the beneficial effects of the antioxidants become actually nocive in cancer therapy has been discussed in some relevant and recent reviews [15], or articles concerning (only) certain specific areas, such as bladder cancer development [16], or ROS- (reactive oxygen species-) based nanomaterials [17], but the exact role of antioxidants and the ambiguity of the oxidants-antioxidants interplay remain unclear. The current study is aimed at offering a comprehensive picture of oxidative stress implication in the various cancer developments and subsequently summarizing the most relevant findings of the anticancer role of all classes of antioxidants, presented here according to their conventional classification. The strategies to improve the efficacy of antioxidants in the clinical context have also been discussed. Data on the antioxidants and ROS controversial or contradictory roles in cancer, illustrative for the antioxidant paradox, the double-faceted impact in tumoral processes, are presented in the final part of this review.

## 2. Free Radicals, Oxidative Stress, and Cancer

### 2.1. Biology of Free Radicals

In the mid-1950s, Gerschman et al. were among the first to suggest that the deleterious effects of O_2_ on the body could be attributed to the formation of oxygenated free radicals [18]. Inspired by their work, Denham Harman, the former chemist of the Shell Company, gave a hypothesis in 1956 famously known as “free radical theory of aging,” postulating that aging and associated diseases are the consequence of a progressive alteration of cellular constituents under the effect of free radicals and ROS [19, 20]. In 1972, Harman extrapolated his theory to the “mitochondrial theory of aging,” proposing that ATP synthesis in the mitochondria involved the electron transfer along a series of membrane multienzyme complexes until their ultimate recovery by molecular oxygen. During this transfer, reactive oxygen derivatives were produced that could generate mitochondrial and nuclear DNA mutations and damage the cellular constitutive proteins. These alterations in turn would cause other mutations promoting the synthesis of other ROS and thus the accumulation of free radicals in the affected cells. Today, the mitochondrial theory is privileged in the explanation of aging phenomena [21]. Harman's point of view was reinforced in 1969 when McCord and Fridovich isolated from human red blood cells the first enzymatic defense system against the superoxide anion produced by univalent reduction of the oxygen, the superoxide dismutase [22].

The discovery that the human body was capable of synthesizing free radicals has thereafter been the starting point for establishing the biological reality of free radicals and their possible pathophysiological roles. The most common radical species in biological systems are the oxygen-derived radicals also known as the reactive oxygen species (ROS). The ROS include oxygenated or primary free radicals such as superoxide anion (O_2_^·-^), hydroxyl radical (OH^·^), or nitrogen monoxide (NO^·^) and also nonradical highly toxic derivatives such as hydrogen peroxide (H_2_O_2_), peroxide anion (O_2_^2-^), and peroxynitrite (ONOO^−^) [23]. The ROS, a natural consequence of aerobic metabolism, are synthesized during certain vital processes at a reasonable dose, their concentration being regulated by the balance between their rate of production and their rate of elimination by antioxidant systems [24–26]. When the balance between prooxidants and antioxidants is maintained, the ROS play a positive physiological role in the normal functioning of the cells, particularly in the phagocytosis process or the programmed cell death (apoptosis) [27–30]. However, when the homeostasis of oxygen becomes imbalanced in the prooxidant direction, it leads to a pathological state known as oxidative stress [31, 32]. Oxidative stress is a risk factor linked with mitochondrial dysfunction for numerous pathophysiological conditions in which cells are subjected to an endogenous or exogenous production of oxygenated free radicals that exceeds their antioxidant capacities and directly damage on contact macromolecules, including lipids, proteins, and DNA [33].

The lipid structures and in particular the cell membranes are the first targets of the ROS because of their richness in unsaturated fatty acids [34–36]. ROS reactions with membrane lipids concretize in lipid peroxidation processes in cascade. Normally, the primary products of lipoperoxidation, the lipid hydroperoxides (LOOH), are detoxified through the enzymatic pathway of glutathione S-transferases (GST); however, when homeostasis is disturbed, the lipoperoxides escaping this detoxification led to toxic aldehydes; the best known of which is malondialdehyde (MDA) a highly reactive and mutagenic dialdehyde; the potential mutagen of MDA was detected in bacterial and mammalian cells [36–38]. MDA forms stable bonds with -NH_2_ amine groups of biomolecules (proteins, phospholipids, or nucleic acids) to produce inter- and intramolecular bridges of amino-3-iminopropene and structural modifications, and thus, it diffuses easily and can reach the cellular nucleus. At this level, MDA can bind with the nucleotide bases, guanine and cytosine, creating bridges between the DNA strands that will lead to the cessation of the replication process or induce mutagenic effects [39]. In addition, the structures induced by MDA are recognized as nonautonomous by the immune system, resulting in an autoimmune response [40, 41].

Another major toxic by-product generated by lipid peroxidation is 4-hydroxy 2-nonenal (HNE) [42, 43]; the higher toxicity of 4-HNE can be explained by its rapid reactions with thiols and amino groups; 4-HNE formed in cells will modify proteins via Michael addition by reacting with compounds having a thiol group (GSH, Cys, and coenzyme A) [44, 45], but also, by the formation of adducts with three different side chains, by its reaction with the -SH groups of the cysteines, and by the formation of Schiff bases with the -NH_2_ groups of the amino acids lysine and histidine [46, 47]. These adductions of HNE proteins modify and cause protein cross-linking and induce carbonyl stress, which contributes to the promotion of cytotoxic events such as cell growth arrest, mitochondrial dysfunction, apoptosis, and necrosis by modifying cellular proteins and nucleic acids, causing microinflammation that can damage neurons and to the progression of chronic schizophrenia, an Alzheimer's pathogenesis [48–51].

Carbonyl stress may also contribute to the progression of cancer; the concentrations of advanced glycation end-product (AGE) marker of the carbonyl stress were found to be higher in the serum of breast cancer patients in the early stage of the disease, but also with advanced breast cancer (stage III and IV) [52]. HNE also forms adducts with DNA, reacting with the -NH_2_ group of deoxyguanosine, resulting in DNA damage and mutagenic effects [53]. Not surprisingly, 4-HNE is considered a second toxic messenger of free radicals; it is found to be involved in a variety of pathologies, especially in humans, including atherosclerosis, Alzheimer's disease, Parkinson's disease, cirrhosis of the liver, and several cancers [54, 55]. Numerous pathologies are associated with lipid peroxidation, such as neurodegenerative diseases (Alzheimer, Parkinson), diabetes, cancers, inflammatory diseases, or aging [56–59].

Proteins also undergo direct and indirect damage following their interactions with the ROS, which are affecting their function by causing conformation modifications, fragmentations of the peptide chain, aggregation of cross-linked reaction products, changes in electrical charges, and increased susceptibility to proteolysis, resulting in loss of protein enzymatic activity and loss of function of receptor transporting proteins [37, 61].

The sulfur amino acids, such as methionine and cysteine, are particularly vulnerable to oxidation. Methionine is converted into methionine sulfoxide, then methionine sulfone that is heavily involved in neurodegenerative pathologies [61, 62]. The oxidation of the thiol group of cysteine (-SH) generates the sulfenic acid (-SOH) leading to the formation of S-S disulfide bridges or of sulfinic (-SO_2_H) and sulfonic acid (-SO_3_H) [60]. Tyrosine forms 3,4-dihydroxyphenylalanine; the nitration of this residue by peroxynitrite generates 3-nitrotyrosine detected in the plasma of diabetic subjects [64]. The basic amino acids lysine, arginine, and histidine constitute, in contact with free radicals, carbonyl derivatives such as 2-oxohistidine, *α*-aminoadipic semialdehyde, and glutamic semialdehyde. Carbonyl proteins are considered markers of the oxidative stress of proteins, and the increase in their rates is observed in a number of pathological conditions such as neurodegenerative diseases, muscular dystrophy [65], cataractogenesis, rheumatoid arthritis [66], and diabetes [67].

ROS from endogenous or environmental sources also pose a threat to the integrity of the genome by causing irreversible cellular changes in genomic components especially point mutations, deletions, gene rearrangements, and amplification [68]. The OH radical is the most important inducer of DNA oxidation reacting directly with all DNA components, such as purine and pyrimidine bases and the skeleton of deoxyribose sugar, and generating single-strand and double-strand breaks, abasic sites, and inter- and intrastrand covalent bonds as well as with the proteins and modified bases [69, 70]. Among the DNA bases, which are particularly sensitive to oxidation reactions, the most widely studied are the guanines as these have the lowest ionization potential and therefore constitute the main target of ROS [71, 72]. The ROS interaction with guanine will produce 8-oxo-7,8-dihydroguanine (8-oxodG) molecules by type I and type II photosensitization reactions [73, 74]. 8-oxodG has a high mutagenic potential leading to G : C to T : A transversions that have been observed in oncogenes and tumor suppressor genes (such as p53 gene), known to play an important role in carcinogenesis [39, 75, 76]. The accumulation of 8-oxodG was detected during liver carcinoma [77, 78]. 8-oxo-20-deoxyguanosine is also reported in the early stages of carcinogenesis [79].

### 2.2. Involvement of Oxidative Stress in Carcinogenesis

**Figure 1:**
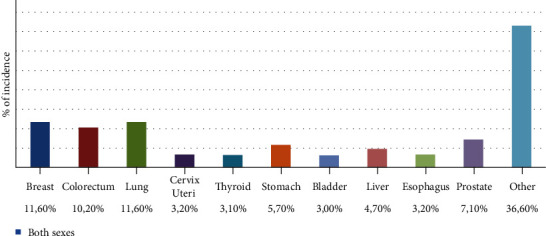
The distribution of cases for the 10 most common cancers in 2018 for both sexes (source: GLOBOCAN 2018 [83]).

It is now well established that the uncontrolled production of reactive oxygen species and the products resulting from their reactions with biomolecules and cells contribute to the etiology of several pathologies, among which the most reported is cancer [25, 80, 81]. Carcinogenesis begins with genetic alterations, in which a very important role is played by high oxidative states [82]. The most frequent invasive cancers, according to GLOBOCAN 2018, an online database providing worldwide estimates of specific cancer incidence, are of prostate, breasts, lungs, gastric, and colorectal (Figure 1).

#### 2.2.1. Colorectal Cancer

Colorectal cancer is the third most common neoplastic disease in the world, and the fourth major cause of mortality [84]. The colon and rectum are permanently exposed to an uncontrolled rate of ROS from endogenous and exogenous sources that lead to the disruption of intestinal homeostasis and can contribute to an increased risk of cancer [85, 86]. Numerous studies have reported increased levels of phospholipase A₂ (PLA2) and myeloperoxidase (MPO) enzymes, associated with the production of high concentrations of free radicals, including RNS and ROS as well as increased levels of MDA and 4-HNE, major products of lipid peroxidation in patients with CRC [87–89]. 8-Oxo-7,8-dihydro-2′-deoxyguanosine, 8-hydroxydeoxyguanosine (8-OHdG), 2-hydroxyadenine, and 8-hydroxyadenine which resulted from DNA oxidation were 2-fold higher in colorectal cancers than in normal mucosa [90]. Similar results were detected in urine sample and peripheral blood leukocytes; a significant increase of 8-oxoGua and 8-oxodG excreted daily in urine additionally to 8-oxodG in leukocytes DNA was reported in colorectal cancer compared to healthy control [91].

#### 2.2.2. Breast Cancer

A study conducted in western Algeria covering 62 breast cancer patients compared to 21 cancer-free controls, evaluating the levels of free oxygen radicals and antioxidants via FORD (free oxygen radicals defense) and FORT (free oxygen radicals test) tests, obtained significantly modified FORT/FORD ratio with increased FORT values and decreased FORD for the cancer patients [92]. The upregulation of oxidative stress markers and downregulation of the antioxidant defense system are considered one of the factors that correlate with the initiation and maintenance of breast cancer progression (Table 1) [92, 93]. DNA in breast cancer was reported to contain high concentrations of base modifications, products of DNA oxidation such as the 8-hydroxydeoxyguanosine, which seem to be involved in breast cancer [94–98]. High levels of urinary 8-OHdG have been detected in women with breast cancer, and the value of 8-OHdG becomes more significant in the later stage of cancer, suggesting that ROS may play an important role in the early carcinogenesis [99]. The ROS are also signaled in the architectural distortion of the breast epithelium inducing fibroblast proliferation, hyperplasia of the epithelium, cellular atypia, and breast cancer [100].

#### 2.2.3. Prostate Cancer

There is strong evidence that ROS from endogenous or exogenous sources are associated with the proliferation of prostate cancer cells [101–104]. The prostate epithelium of patients with this type of cancer is subjected to more intense oxidative stress as compared to men without the disease [102]. This increase in the generation of ROS is seemingly the result of the overexpression of the various isoforms of NAD(P)H oxidases (NOX), demonstrated in the cancer cells of the prostate [105, 106]. To identify the levels of ROS and the role of the extramitochondrial ROS generator, in the NOX system in prostate cancer cells, Kumar et al. used three cell lines (LNCaP, DU145, and PC3), normal prostate cell cultures (WPMY1, RWPE1, and primary normal epithelial cell cultures), and agents (the NOX inhibitor diphenyliodonium and the antioxidant N-acetyl-L-cysteine) that neutralize the effect of ROS and block their extramitochondrial generation. The results showed that NOX inhibition and ROS neutralization induced growth arrest and blocked proliferation of prostate cancer cell lines and that ROS generation was directly proportional to the aggressive phenotype [107]. A significant decrease in GPx–selenoenzyme and GSH in PC prostate cancer patients has been very well documented by Zachara et al. and Szewczyk-Golec et al., in tissue and serum compared to the control [108, 109]. These results showed that the lack of GPx defense system accompanied the increase of OS and thus the development of pathological prostate conditions. According to Miyamoto et al., the loss of GPx activity was associated with oxidative stress, through glycoxidative stress and two steps of nitroxidative stress, reversible by nitrosation of a selenium moiety, and irreversible by formation of a sulfur-seleno bridge between Cys91 and Sec 45 [110, 111].

#### 2.2.4. Lung Cancer

Lung cancer is a leading cause of cancer-associated mortality worldwide, causing 1,000,000 deaths per year, due to a poor prognosis, high resistance to treatment, and poor survival rate [112, 113]. In lung cancer, oxidative stress is found to be a consequence of the disease as well as cooccurrence with smoking, which appears to be a major cause of lung cancer with 90% and 70-80% of cases of lung cancer men and women, respectively [114–118]. In a study conducted by Xu et al. 8-OHdG was elevated in mice treated with nitrosamine 4-(methylnitrosamino)-1-(3-pyridyl)-1-butanone (NNK) major components of tobacco that are associated with the etiology of lung cancer [119]. Smoking therefore contributes to the supply of oxidants not only by exogenous means but also by endogenous means through the induction of chronic inflammation of the respiratory tract with accumulation and activation of leukocytes. This activation leads to high concentrations of ROS/RNS and induces genetic mutations, incessant damage to DNA, inactivation of apoptosis, regulation of growth factors and cytokines, and activation of growth supporting genes, ultimately leading individuals to a high risk of lung cancer [114, 120–123]. The exogenous oxidants cause lung injury by a number of mechanisms, including depletion of glutathione and other antioxidants, initiation of redox cycling mechanisms, improvement of respiratory thrust in neutrophils and macrophages, inactivation of protease inhibitors such as a1-antitrypsin inhibitor, and direct damage to lipids, nucleic acids, and proteins [124]. The reaction of NO with ROS altered the function of proteins by nitridation or induced structural alterations of DNA, including single- or double- strand DNA breaks, purine, pyrimidine, or changes in deoxyribose and reticulation of DNA; damage to DNA induced either stopping or induction of transcription, induction of DNA pathways signal transduction, replication errors, and genomic instability, contributing to the progression of pulmonary carcinogenesis [125].

#### 2.2.5. Gastric Cancer

Gastric cancer is the fifth leading cause of cancer. It has a relatively rare occurrence in individuals less than 40 years of age [83]. However, the highly aggressive and heterogeneous nature of these cancers has made them the third leading cause of deaths in cancer patients, globally [126]. The stomach is continuously exposed to ingested pollutants and carcinogens. The ROS are also produced in large amounts due to bacterial metabolism and food digestion in a highly acidic environment. The cytoprotective mechanism of the gastric system and ingested antioxidants naturally prevent the above factors from affecting the stomach. However, several internal and external elements including lifestyle disorders, exposure to pollutants, and prevalence of *Helicobacter pylori* infections can cause a disbalance in the cytoprotective mechanisms and lead to production of highly reactive and cytotoxic oxidative products like ketones, epoxides, and reactive aldehydes. These compounds trigger the formation of DNA adducts including malondialdehyde-deoxyguanosine, advanced lipid peroxidation end products, and AGEs and promote the prognosis of gastric cancer [127]. Although genetic factors predispose to development of gastric cancer, the above factors play a significant role in its development and prognosis [128].

Unlike other types of cancers, the correlation between dietary factors and reduced risk of development of gastric cancers is well established by the World Cancer Research Fund/American Institute for Cancer Research (WCRF/AICR). The antioxidant activity of flavonoids like quercetin and naringin present in apples and grape fruit, respectively, can inhibit carcinogen-activating enzymes in case of gastric cancers. The metabolism of toxins into carcinogens due to the expression of CYP1A1 (cytochrome P450 enzyme) is also inhibited which prevents DNA damage [129]. In general, it is known that the phytochemicals downregulate the *β*-catenin phosphorylation to enhance the apoptotic pathways in events of gastric cancer and simultaneously upregulate the AMPK pathway to support cellular homeostasis [130]. A recent study indicated a strong expression of the MUC4 gene in human gastric cancers. It was reported that the antioxidant alpha-lipoic acid inhibits the binding of STAT3 to the MUC4 promoter region to suppress the MUC4 expression and hence significantly reduces the proliferation of gastric cancers [131]. The anthocyanin (delphinidin)-rich extracts of calafate berries also significantly reduces the viability and migration of stomach cancer cells [132].

ROS appear to be involved in the progression of tumor progression [133], and a healthy organism must therefore be endowed with an adequate cellular defense to eliminate these molecules of extreme toxicity. These antioxygenic defense mechanisms are more precisely known as the antioxidants [134].

## 3. Antioxidants in Cancer Therapy

According to Tang and Halliwell, an antioxidant is defined as any substance that slows down, prevents or inhibits the generation of a toxic oxidant, neutralizes those already produced by donating their own electrons, and thereby prevents the harmful chain reactions caused by these oxidants [135]. Under this definition, antioxidants are classified according to their mode of action, their cellular location, and their origin into endogenous, enzymatic and nonenzymatic, and exogenous [136]. In recent years, the topic of antioxidants has become more famous in modern society in cosmetics and nutrition, but the use of antioxidants is at the center of the scientific debate when it comes to prevention or supplementation in oncology therapy, with benefits, but also harmful effects being reported [137].

### 3.1. Antioxidants, a Protection against Cancer

Many researchers have confirmed the involvement of the free radicals and ROS in the pathogenesis of many cancers [81, 138, 139], so it is logical to think that the alternative medicine of antioxidants acts in the prevention of this disease. Beyond their ability to trap ROS, antioxidants exhibit anticancer activities by increasing the immune response, stimulating cancer recessive genes, decreasing oncogene expression, or inhibiting tumor angiogenesis [140]. Below, we provide a summary of the strong evidence supporting the protective effect of the different classes of antioxidants.

### 3.2. Endogenous Enzymatic Antioxidants

#### 3.2.1. Superoxide Dismutase (SOD)

The superoxide dismutases, which make a class of metalloproteins, are one of the most effective antioxidant enzymes. In mammals, there are three isoforms, each of which has a metal ion as a cofactor: the cytosolic Cu/Zn-dependent SOD, the manganese mitochondrial SOD, MnSOD, and the extracellular Cu/Zn-SOD [141]; these differ in the chromosomal location of the gene, the nature of the active site metal, their quaternary structure, their cellular localization, and other characteristics [142], but they all catalyze the dismutation of the highly reactive superoxide anions to oxygen and the less reactive (but potentially toxic) hydrogen peroxide [143, 144]. SOD must therefore work in conjunction with other enzymes such as the iron-cofactor catalase and glutathione peroxidase, to eliminate the formed hydrogen peroxide [145].

There are numerous studies that have showed that the expression and enzymatic activities of all the three SOD isotypes are at a significant decrease across the majority types of cancers: CuZnSOD (SOD1) activity is decreased in breast carcinoma [146], gastric adenocarcinoma [147], and hepatocarcinogenesis [148] and MnSOD (SOD2) expression is downregulated in the ductal carcinoma tissues in patients with breast cancer [149] and skin cancers [150] and displays low levels which correlates with pancreatic cancerous tumor growth [151] and other primary ovarian and prostate tumors [152]. Extracellular SOD (SOD3) expression is downregulated in lung carcinomas [153] but also in prostate pancreatic, thyroid, or colorectal cancers according to Griess et al. [154]. Moreover, Bonetta [155] provides in a recent descriptive review evidence of multiple studies the relevance of MnSOD gene therapy [156] or of synthesized SOD mimetics in cancers [157, 158].

The diminished activity of SOD is associated with the destructive effects of O_2_^·−^ superoxide which by way of deprotonation of serine or threonine residues contribute to the accelerated rates of phosphorylation in many oncogenic signaling processes with subsequent antiapoptotic effect and tumoral progression [154].

The downregulation of MnSOD expression has been associated with changes in gene regulation mechanisms, such as altered DNA methylation patterns that involve abnormalities in transcription factor binding, such as the widely studied Sp1/Sp3 [159, 160], or decreased histone acetylation [161]. The aberrant DNA methylation, which can occur as either global hypomethylation in the early phase of neoplasia or regional hypermethylation of normally unmethylated CpG sites has been previously showed to be induced, among others, by reactive oxygen species and their derivatives DNA damage [162–164]. On the other hand, Robbins and Zhao [159, 165] describe the antitumoral effect of MnSOD overexpression in a multistage skin carcinogenesis mouse model that was found in a previous study by [150], as involving suppression of activator protein-1 (AP-1) a transcription factor regulating oncogenic signaling. At the same time, the dimer AP-1 function depends on redox-sensitive Jun or Fos-type subunits and their phosphorylation states, which suggest the antioxidant capabilities of MnSOD in tumor suppression [159].

In light of these multiple results, the importance of SOD as a tumor suppressor seems well justified, in particular of MnSOD, whose active role in protecting mitochondria against increased oxidative stress may prevent defects in mitochondrial function that would lead to the development and progression of cancer [166].

However, there is yet a “puzzling dichotomy” on SOD role in cancer [167], as clinical and experimental studies bring enough evidence on unexpectedly high levels/activity of SOD in tumoral tissues [168–170]. In the most recent study on the antioxidant enzyme dichotomy, Gaya-Bover et al., assessed by western blot the levels of MnSOD, CuZnSOD, CAT (Catalase), GPx, and other proteins both in tumor and nontumor adjacent tissue from colorectal cancer patients found that nontumor adjacent tissue of had higher levels of antioxidants enzymes that detoxify H_2_O. However, tumor tissues showed higher levels of MnSOD and acetylated MnSOD that actually increased in stages II and III compared to stage I of malignancy and precancerous stage [171]. Palma et al. state that SOD2 undergoes a change of roles from a suppressor of tumor initiation to actually promoting tumor progression towards more malignant phenotypes, once the disease is established [167]. This observation is further contextualized by Kohan et al., when referring to the importance of aerobic glycolysis in tumor cell survival [168]. Thus, the MnSOD upregulation suppresses mitochondrial oxidative phosphorylation and leads to the activation of AMPK (AMP-activated protein kinase) that triggers the Warburg effect switch to glucose metabolization via glycolysis [168]. The glycolytic phenotype switch of the malignant cells is regarded as an adaptive advantage that decreases dependence on mitochondrial respiration and enables them to proliferate and invade into the peritumoral normal tissue [172].

#### 3.2.2. Catalase (CAT)

Catalase, a heme oxidoreductase, strongly expressed in the liver, erythrocytes, and lungs [173] and located mainly in the peroxisomes [174] is composed of four polypeptide chains and contains iron atoms within the heme, which constitute the active sites of the protein [175]. The dismutation of the hydrogen peroxide by catalase happens with the heterolytic cleavage of the O-O bond of the hydrogen peroxide by the iron atoms of the heme group, thereby creating a water molecule and a highly oxidizing FeIV=O bond [143, 144]. The latter can oxidize a new molecule of hydrogen peroxide, generating dioxygen.

Catalase uses only H_2_O_2_ as a substrate and functions when it is present at high concentrations above physiological conditions; it does not eliminate all of the hydrogen peroxides and is quantitatively less effective than the glutathione peroxidase [176].

The overexpression of catalase in breast cancer cells MCF-7, a human-derived breast cancer cell line, not only impaired the proliferation and migration of the cancer cells but also improved sensitivity to anticancer treatments like doxorubicin, cisplatin, and paclitaxel when compared to parent cells and raised the resistance to the prooxidant combination ascorbate and menadione (Asc/Men) [177]. The elevated catalase activity confers strong protections against cancer by reversing the malignant phenotype via decreasing the cellular levels of ROS production (mainly H_2_O_2_) [177]. The high activity of CAT and when combined with chemotherapeutic drugs especially cCisplatin, 5-fluorouracil, and hydroxyurea correlate with decreased aggressiveness and suppress the proliferation of small lung cancer cell lines—A549 compared to untreated cells [178]. CAT treatment leads to disturbing the antioxidant defenses pathway and then a high production of intracellular H_2_O_2_ that could be responsible for the transcription factors NF-*κ*B inactivation associated with promotion of tumorigenesis in A549 cells [178]. Bracalante et al. demonstrated that overexpression of catalase downregulates cell proliferation and reversed the amelanotic phenotype in clones A7 of the human A375 amelanotic melanoma cells compared with the parental cell lines [179]. A7 increased basal levels of hydrogen peroxide and as a result upregulated genes of the antioxidant system (AOS) induced and increased the ability of these cells to respond to oxidative stress, and then reduced the ROS levels, leading to less aggressive tumor cells [179].

The transfected RASMC vascular smooth muscle cells with AdCAT construct containing the human CAT (50-100-fold excess) reduced proliferation and DNA synthesis with increased spontaneous apoptosis through a COX-2-dependent mechanism compared with the corresponding parental cells [180]. The cell survival and the apoptosis resistance in RASMCs were maintained by H_2_O_2_; it is a downregulation by catalase-suppressed malignant phenotype [180, 181].

#### 3.2.3. Glutathione Peroxidase (GPx)

Glutathione peroxidase, a selenium-dependent enzyme, present in extracellular fluids, cytosol, and mitochondria [182], was first identified by Mills in 1957 for its antioxidant activity [183]. Unlike catalase, glutathione peroxidase reduces a wide variety of organic hydroperoxides (ROOH) and neutralizes H_2_O_2_ using the reduced glutathione monomer (GSH) as a proton donor cofactor (H^+^), which is subsequently converted into oxidized glutathione (GSSG); ultimately, the reactions lead to the formation of assimilable molecules, a cysteine or disulfide bridge, water molecules, and alcohol [144, 184].

Increased GPx levels were observed in many tumor cells, such as the oral squamous cell carcinoma (OSCC) [185], lung [186], colorectal [187], breast [188], and brain tumor [189] patients, compared with healthy controls. This can be explained by the fact that the tumor has high levels of oxidative stress; the body increases then the antioxidant system levels to compensate the increased levels of ROS as a natural defense against cancer [190]. Adenovirus-mediated human glutathione peroxidase gene (AdGPx) overexpression reduced the growth of pancreatic cancer cells in vitro and in vivo models. In vitro AdGPx enhanced the tumor-suppressive effect of AdMnSOD; their combination slows the MIA PaCa-2 cells growth by 71% compared to parental cells. While after AdMnSOD and AdGPx injection into nude mice either alone or in combination showed a greatest effect by decreasing the tumorigenicity of pancreatic tumor cells [191]. The GPx increase is related to a H_2_O_2_ increase or other hydroperoxides caused by overexpression of MnSOD; the GPx activity neutralizes the increased hydrogen peroxide levels caused by overexpression of MnSOD to maintain intracellular homeostasis [191–194]. In addition, a high GPx expression drastically impeded tumor growth of weakly tumorigenic L929 fibrosarcoma cells injected subcutaneously into nude mice and increased the sensitivity of strongly tumorigenic B16BL6 melanoma cells to an angiodestructive therapy [195]. GPx4 inhibits tumor angiogenesis and malignancy through blocking eicosanoid synthesis, including COX-2, prevented TNF-mediated activation of cytokine-driven NF-*κ*B and prostaglandin PGE2 production and abrogated PGE2-dependent COX-2 expression [195].

### 3.3. Endogenous Nonenzymatic Antioxidants

#### 3.3.1. Bilirubin

The final product of the catalytic degradation reaction of heme and hemoglobin proteins by reticuloendothelial cells in the presence of two enzymes, heme oxygenase and biliverdin reductase [73], is considered a very important physiological antioxidant [196]. Bilirubin is remarkable for its powerful antioxidant potential against peroxyl radicals (ROO^·^) and hydrogen peroxide [197, 198].

Due to its double action as a powerful antioxidant and as a prooxidant, bilirubin has been shown to have anticancer activity in both in vitro and in vivo, depending on the concentration and the cell type exposed to this pigment [199–202]. Emerging studies have suggested that serum bilirubin levels were negatively correlated with cancer risk and to cancer mortality [203–207]. In vitro, a 48 h treatment with a concentration of 10 *μ*g/mL of bilirubin induced antiproliferative behavior in TMK-1 human gastric carcinoma cell line with a decrease close to 50% in cell population versus control and increased by more than twofold the intracellular radical levels after 6 h-24 h of incubation while no change was observed in control [202]. Bilirubin also caused a dose-dependent decrease (0–50 *μ*M; for 48 h) in cell viability of colon adenocarcinoma cell lines [208]. The defense mechanisms by which bilirubin exhibits antitumor properties can be explained by the induction of prooxidant effects, apoptotic action, arresting cell cycle, and disrupting mitochondria dysfunction [202, 208].

In vivo, an intraperitoneal injection of 25 mg/kg of bilirubin in BALB/c nude mice bearing HRT-18 colon cancer xenografts increased plasma bilirubin levels around 35–40 mM and dramatically slower the tumor growth when compared to the control phosphate-buffered saline. The bilirubin exert anticancer activity by halting cell cycle progression at G1 phase through inducing p53 and the activation of MAPK/ERK-dependent p27 pathway which inhibits the activation of cyclin E/CDK2 and/or cyclin D/CDK4 complexes [209]. The same study describes an in vitro bilirubin dose-dependent model using multiple cell lines; at a lower dose (5 mM), bilirubin inhibits cell proliferation of hepatocellular carcinoma HepG2 and NIH/3T3 immortalized mouse; the antiproliferative behavior of MDA-MB 231 human mammary gland ductal carcinoma cells, pancreatic PANC-1, malignant melanoma WM 266.4 including colonic HRT-18 studied in vivo can only be obtained in a concentration ≥ 25 *μ*M. The antioncogenic effect of bilirubinin was found to be partially dose dependent; at a high concentration, it acts as a proapoptotic factor in the tumor environment [209].

#### 3.3.2. Coenzyme Q_10_ (CoQ_10_)

Coenzyme Q_10_, known as ubiquinone, is a hydrophilic compound essential for the transport of electrons in the mitochondrial oxidative chain [210, 211, 298]. It is found in cell membranes, plasma, and lipoproteins where it acts as an antioxidant by trapping RO_2_^-·^ radicals, thus inhibiting lipid peroxidation [198].

Because of its double role as a potent antioxidant molecule and as redox status at the mitochondria [213], the coenzyme Q has been proposed to have therapeutic potential for the treatment of several types of cancer [214–216]. Coenzyme Q_10_ deficiency has been reported in many cancer patients. A decreased mean value of blood vessels of coenzyme Q_10_ have been found in patients with breast and myeloma cancer compared to healthy controls [217]. In vitro, the response of PC3 human prostate cancer cells towards CoQ_10_ was markedly different compared to PNT2 human prostate nonmalignant cells. A 24 h incubation of cancer cells with different concentrations of coenzyme Q_10_ (50, 100, and 250 *μ*M) significantly lowered cancer cells grow PC3 with no change on PNT2 noncancer cells compared to the control; the results showed also a greater free radical generation in PC3 [213]. Apparently, the abundance of ROS can explain the antitumor potential of CoQ_10_; the cancer cell killing was found to be on Ros-induced cytotoxicity dependent through apoptosis, necroptosis, and autophagic cell death [218]. At high concentration, the CoQ_1_, an analog of CoQ_10_, showed remarkable antiproliferative effects in HepG2 human hepatocellular cancer cells. Although CoQ_1_ was slightly more toxic to Hep G2, the anticarcinogenic effect of CoQ_1_ can be attributed to its antimetabolite and cytotoxic activities, which disrupt normal biochemical reactions [219, 220].

Another analog of CoQ_10_ appears to be involved in the antitumor defense, the coenzyme Q_0_ (2,3-dimethoxy-5-methyl-1,4-benzoquinone) decreased cell viability of A549 adenocarcinoma human alveolar basal epithelial cells and show a strong cytotoxicity towards both breast cancer cell lines MDA-MB-231 by arrest of G0/G1-phase cell cycle and S-phase cell cycle arrest in SKBr3 cells by inducing apoptogenic phenotype and promoting cell death [221, 222]. The anticancer efficacy and the cytotoxicity of the CoQ analogs can be also chain length dependent; the antiproliferative activity of CoQs was found to be negatively correlated with the length of CoQ's isoprenyl side chain [222].

In vivo supplementation with CoQ_10_ (0.4 mg/kgG1/day) has a therapeutic effect against hepatocellular neoplasia HCC induced by toxic agents such as trichloroacetic acid (TCA) in male Sprague-Dawley rats; these compounds exert a carcinogenic action by increasing oxidative stress, lipid peroxidation, and inflammation. The administration of CoQ_10_ ameliorated the histopathological dysplastic changes, prevented the reduction of GSH and SOD activity, and significantly attenuated the expression of the nuclear factor-*κ*B signaling pathway which enhances the transcription of TNF, iNOS, and COX-2 genes; CoQ_10_ reduced significantly both hepPar-1 and AFP immunomarkers of tumor in the liver cells of rats [223]. The CoQ_10_-glucan synergies showed a high metastatic activity in Balb/c mice implanted with Ptas64 mammary carcinoma; the volume of tumors (mm^3^) after CoQ_10_-glucan supplementation was two times smaller than supplementation with glucan alone; the CoQ_10_ seems to reinforce the capacity of glucan to half the development of tumor through the stimulation of innate immune cells such as macrophages, dendritic cells, granulocytes, and natural killer cells and activation of mitogen-activated protein kinases (MAPKs) and nuclear transcription factor NF-*κ*B p6512 [224, 225].

#### 3.3.3. Melatonin

Melatonin or N-acetyl-5-methoxytryptamine is a neurohormone mainly secreted by the pineal gland, but also in extrapineal organs including the retina, gastrointestinal tract, skin, bone, marrow, and lymphocytes, with an insignificant systemic contribution, and is synthesized from N-acetylation, followed by methylation of serotonin in the absence of light through the use of a methylation factor. It is synthesized by N-acetylation, followed by methylation of serotonin in the absence of light through 2 transferases: aryl-alkylamine-N-acetyltransferase (AANAT) and 5-hydroxy-indole-methyltransferase (5 HIOMT). Its first role was discovered in interaction with its organ of origin the pineal gland and was linked to its neuroendocrine function, in particular to the hypothalamo-pituitary-gonadal axis. A second obvious functional role of melatonin is involved in the synchronization of the sleep/wake cycle (chronobiotic role) [226]. Besides these functions, melatonin has been shown to act as a free radical scavenger and a potent endogenous antioxidant [227]. In *in vitro* cell-free systems, Tan et al. showed that melatonin (IC50 = 21 *μ*M) quenches 50% of the adducts (DMPO-· OH) generated following exposure of H_2_O_2_ to ultraviolet light, five times more than GSH (IC50 = 123 *μ*M) and thirteen times more than mannitol (IC50 = 283 *μ*M) [228]. A structure-activity relationship was thus established, among the 3 indoleamine analogues of melatonin, 5-hydroxytryptamine (5-HT) lacking the methyl group in the 5-OH position of the indole ring and the acetyl group linked to the N position of the side chain; in N-acetyl-5-hydroxytryptamine (NA-5-HT) lacking the methyl group in the 5-OH position and 5-methoxytryptamine (5-MT) lacking an acetyl group in the N position of the side chain, only 5-MT reduced the formation of the adduct DMPO-· OH adduct with an efficiency lower than 60% of that of melatonin at high concentration [229]. These data show that the methyl group located at the 5-OH position of the indole ring of methionine gives it an important capacity for its hydroxyl radical trapping function, while the N-acetyl group produces a synergistic action. The melatonin (IC50 = 1.5 mM) has also been found to neutralize directly the nitric oxide generated by 1-hydroxy-2-oxo-3-(N-methyl-3-aminopropyl)-3-methyl-1-triazene (NOC-7) in a dose-dependent manner and more effectively than N-acetylserotonin (IC50 = 20 mM), 5-hydroxytryptophan (IC50 = 15 mM), L-tryptophan (*IC*50 = 40 mM), and serotonin (IC50 = 8 mM), with a large biomolecular rate constant of 3.0 × 10^7^ m^−1^ s^1^. This potent ability to lower NO- concentrations can be explained either by direct scavenges or incapacitating its production while inhibiting its synthesis by lowering the activity of the prooxidative enzyme NO synthase [230]. This free radical scavenging ability extends to other radicals as well. Melatonin (ORAC peroxyl = 2.04) was found to be twice as effective as Trolox (ORAC peroxyl = 1.00) and vitamin C (ORAC peroxyl = 1.12) and three times more effective than reduced glutathione (ORAC peroxyl = 0.68) in detoxifying the peroxyl radicals ROO· generated by Azobis (2-methylpropionamidine)dihydrochloride (AAPH) [231]. Additionally ORAC-OH shows also that melatonin was 2-fold efficient than Trolox and 8-fold than dopamine, with GSH as a prooxidant [232]. Adding to its radical scavenging ability, melatonin also stimulated the levels of several antioxidative enzymes, including SOD, glutathione peroxidase, glutathione reductase, and catalase [233]. Melatonin administration (10 mg/kg) increased superoxide dismutase, GSH system like GPx, and catalase activities with a tissue-dependent degree and reduced MDA+4 HDA lipid peroxidation markers in the kidney, liver, and brain homogenates compared to the group of rats receiving twice weekly for 3 months 10 mg/kg of benzo(a)pyrene B(a)P alone [233]. A similar response was observed in xenografts (induced by the CT26 cell line) in BALB/c mice. Melatonin showed radioprotective action at 20 mg/kg in the lung and cardiac tissues after a single 5 Gy gamma ray exposure by stimulating GPx and SOD activities and depleting MDA level [234]. Melatonin not only upregulates antioxidant enzymes but also downregulates prooxidant enzymes such as myeloperoxidase, nitric oxide synthase, and eosinophil peroxidase [235, 236]. Also, AMK melatonin metabolites generated via the kynuric pathway acted as antioxidants and thus slowed down the prooxidative enzyme nitric oxide synthase (iNOS) [237, 238]. Another benefit of melatonin was recorded in the brain of rats treated with kainic acid, a neuronal excitotoxin. Melanonine played a protective role against DNA damage, by reducing the increase in oxidative marker 8-hydroxy-2-deoxyguanosine (8-OHdG) in both the frontal cortex and hippocampus. The proposed mechanism through which melatonin protects DNA from KA damage may be associated with nitric oxide (NOS) inhibition [239].

Melatonin has many therapeutic properties in the treatment of cancer through several mechanisms, including inhibiting cancer cell proliferation, decreasing oxidative stress, and increasing immune system activity. Liu et al. reported that 2 mM melatonin attenuates the proliferation of the MFC gastric cancer cell line in mice, by upregulating the mRNA expression of the transforming growth factor-*β*1 (TGF-*β*1) in tumor tissues. TGF-*β*1 has been shown to suppress tumor development in a time- and dose-dependent manner, by blocking the cell cycle (G1-phase arrest), via increasing cyclin-dependent kinase (cdks) inhibitor levels [240, 241]. This report was corroborated by Bizzarri et al. At physiological concentrations, the synergy of melatonin with low noncalcemic doses of 1,25-(OH) 2D3 significantly inhibited the proliferation of rat RM4 breast cancer cells in the presence of estrogen by enhancing the TGF-*β*1 effect [242, 243]. In hepatocellular carcinoma, melatonin showed significant tumor growth inhibition both *in vitro* and *in vivo.* The melatonin potentiates sorafenib-induced apoptosis in 3 hepatoma cell lines HepG2, HuH7, and Hep3B via synergistic activation in a dose- and cell type-dependent manner, by enhancing ROS production, proapoptotic genes PARP hydroly and Bax, reducing the amount of mt-DNA in treated cells, and mitophagy-mediated by mitochondria and lysosome colocalization, rising the expression of mitophagy markers PINK1 and Parkin by downregulating heat shock protein 60 (Hsp60) [244]. Similar results were found *in vivo*; melatonine increased the therapeutic potential of mesenchymal stem cells (MSCs) in adult female rats after diethylnitrosamine- (DEN-) induced hepatocellular carcinoma (HCC). Melatonin thwart HCC carcinogenicity by inhibiting oxidative stress designated by the decrease in malondialdehyde levels and the increase in the activities of the enzymes superoxide dismutase, catalase, and glutathione peroxidase; inflammation by downregulating the expression of interleukin-1 beta, nuclear factor kappa B, vascular endothelial growth factor, and matrix metallopeptidase 9 genes; and upregulated expression of metalloproteinase inhibitor 1 gene, and induction of apoptosis indicated by increased activity of cleaved caspase-3 and Bax genes and downregulated expression of antiapoptotic genes Bcl2 and survivin [245]. Moreover, Yun et al. investigated the prooxidant and apoptotic efficacy of melatonin in a wild-type human colorectal cancer cell line (SNU-C5/WT) in a dose-time-dependent manner. The result showed that melatonin lessened SNU-C5/WT viability, through enhancing superoxide generation and reducing cellular prion protein (PrPC) expression, as well as PTEN-induced kinase 1 (PINK1) levels. This advanced mitochondria-mediated apoptosis and endoplasmic reticulum stress. This study highlights the promising strategy to target colorectal cancer [246]. Into the bargain, (20 *μ*g/mL) melatonin and (50 mg/j) capecitabine synergized together and improved survival and tumor growth inhibition *in vivo* in pancreatic tissue of male Syrian hamsters injected with the carcinogen N-nitroso bis(2-oxopropyl) amine (BOP), compared to capecitabine alone; this combination showed an upgrade in the antioxidant enzymes GSH, SOD, CAT, and GSH-Px activities and a retrogression of lipid peroxidation products [247].

### 3.4. Exogenous Antioxidants

#### 3.4.1. Trace Elements

*(1) Selenium*. A mineral micronutrient with strong antioxidant activity, essential for the defense of the body against damage caused by oxidative stress [248, 249], was acquired through diet; selenium is located mainly in the liver, kidneys, blood, brain, heart muscle, and testes. As a cofactor, selenium is incorporated in the form of selenocysteine into selenium-dependent enzymes, such as the GPx; hence, its role in free radical scavenging [250]. Selenium is also an antagonist of many metals (As, Cd, Pb, and Hg) and is therefore likely to modulate their toxicity which causes oxidative stress, disruption of pigment function, and alteration in protein activity [251, 252].

The selenoproteins act as a defensive barrier against reactive oxygen species and inflammatory responses [131]. Several studies have described the potential of selenium in the induction of apoptosis and selective cytotoxicity to cancer cell lines derived from the lung, colorectal, breast, esophageal, stomach, and prostate [132, 253, 254]. Recently, a study suggested that intracellular selenium and cisplatin may act synergistically and modulate the transient receptor potential vanilloid 1 (TRPV1) cation channel. These channels are responsible for the regulation of body temperature and sensing scalding sensory stimuli in events of distress. Hence, to some extent, relief from cancer-associated pain is possible through the above synergistic therapy. Besides, they also reported an increase in apoptotic proteins and a reduction in cell viability in the MCF-7 breast cancer cell line cultures on treatment with selenium as well as selenium+cisplatin. Also, an increase in ROS occurs on the use of chemotherapeutic agents including cisplatin. Selenium being an antioxidant overcomes the side effects of chemotherapy in general [255]. As a part of the recent development in cancer treatment, selenium-based nanobiocomposites of L-asparaginase have also been constructed which act as improved drug delivery systems with intact antioxidant function. Thus, it opens new dimensions to cancer therapy [256].

*(2) Zinc*. Zinc is the second most abundant elemental trace element in the body after iron, mainly found in bones, muscles, and liquids rich in proteins (plasma or cerebrospinal fluid) [257]. With indirect antioxidant property [258], this mineral participates in the structure or the regulation of more than 300 enzymes, in particular the antiradical enzymes involved in the protection against the toxic derivatives of oxygen, such as the superoxide dismutase [259]. Thus, the catalytic complexes that zinc and thiol groups form have been observed to stabilize and protect proteins against oxidation [260]. Zinc is also involved in maintaining the tissue functional levels of metallothionein, a molecule potentially capable of trapping free radicals [261].

Well-established evidence suggests decreased activity of ZIP1 (SLC39A1)-zinc uptake transporter in malignant cells. Although this indicates that zinc deficiency may be a triggering factor for cancer development, more studies are required to sustain the claim with absolute certainty [262]. However, zinc deficiency is strongly associated with esophageal squamous cell carcinoma, prostate cancer, and colon cancer [263]. It is also noted that zinc deficiency prominently causes oxidative DNA damage and simultaneously impairs the DNA repair ability in cells leading to increased frequency of mutations [264, 265]. The anticancer activity of zinc is associated with its significant role in promoting transcription factors for cellular propagation, antioxidant defense system, and DNA repair [263]. In prostate cancer, the modulation of cancerous cell growth is observed in the LNCaP cell-line with increased zinc supplementation. This is associated with the activation of the ERK1/2 phosphorylation and reduction in VHR phosphatase and ZAP-70 kinase levels that together result in antiproliferative activities of zinc [266].

*(3) Copper*. Copper plays multiple roles in the metabolism of carbohydrates, lipids, and proteins, in the maintenance of bone mass and cartilages, and in the synthesis of hemoglobin and also contributes to immune defenses and the fight against the phenomenon of oxidation and aging [267–272]. In this regard, copper is a powerful antioxidant stimulating the activity of SOD, cytochrome C oxidase, and dopamine *β*-hydroxylase [73, 273].

Elemental copper is reported to be a strong modulator for the expression of several growth factors including vascular endothelial growth factor (VEGF), interleukins, and oxidative phosphorylation. Thus, it stimulates angiogenesis, cellular proliferation, migration, and oxidative stress. Hence, the anticancer therapies are largely based on anticopper (copper chelation) treatment approach, especially for metastatic cancers [274]. Copper complexes, on the other hand, show a spectrum of activities depending on the ligands attached to it and have been associated with both mitotic and apoptotic cancer cell deaths. The copper complexes prepared with N-benzyl 2-(diethyl amino) acetamide and 2-(diethyl amino) N-phenyl ethyl acetamide have shown anticancer activity in the U87 and HeLa cell lines [275]. The mixture of dithiocarbamates and copper salts react with cellular copper to form a proteasome that is responsible for inducing apoptosis [276] and also dismutates superoxides into harmless compounds [277]. Thus, they are emerging as promising metal-based cytotoxic agents for cancer therapy.

#### 3.4.2. Vitamins

*(1) Vitamin C*. Vitamin C (ascorbic acid; C_6_H_8_O_6_) is a heat and thermo labile antioxidant [278] that represents the most important line of defense against oxidative stress in extracellular fluids, acting as an antioxidant and cofactor in oxygen-catalyzed hydroxylation reactions [279]. It is a powerful scavenger of the ROS including the superoxide anion, singlet oxygen, H_2_O_2_, NO, and OH [280]. The antioxidant role of vitamin C is based on the inhibition of lipid peroxidation by reducing the oxidized vitamin E after its reaction with lipid radicals, generating the ascorbyl radical, which can be reduced by glutathione-dependent enzymes [281]. Vitamin C protects biomembranes and lipoproteins [282].

Dietary supplementation of vitamin C has been associated with improved patient outcome in advanced cancers of breast and pancreas [283]. Moreover, a synergistic action of ascorbic acid and anticancer agents like etoposide, cisplatin, and doxorubicin has also been reported. In 2015, a study reported selective death of cells with KRAS or BRAF mutations in human colorectal cancer cells. Interestingly, the dehydroascorbate (oxidized form of ascorbate) was found to compete with GLUT1 transporter (for glucose uptake) and reduced to ascorbic acid inside the cancer cells. The increased uptake of DHA and the subsequently increased concentration of vitamin C inside the cells can thus be correlated to death of cells undergoing above mutations [284]. Vitamin C-associated tumor inhibition and reduction in tumor aggressiveness have also been reported in gastric cancers [285].

*(2) Vitamin E*. The liposoluble vitamin E is the most important antioxidant of the hydrophobic interior sites of cell membranes [286]. Under this designation, the tocopherol and tocotrienol families are grouped, with alpha-tocopherol as the most abundant and most biologically active form [146, 287, 288]. This lipophilic antioxidant has the capacity of trapping and neutralizing free radicals such as lipoperoxides, forming nontoxic tocopheryl radicals and interrupting the lipid oxidation chain [73], and also increasing the activity of antioxidant enzymes such as SOD, GPx, catalase, glutathione transferase, and NAD(P)H reductase [289].

Vitamin E has been, most convincingly, identified as a potential adjuvant in the treatment of cancers. Dietary deficiency of vitamin E in generalized populations of Northern Europe and USA and their increased risk of colon and prostate cancer are clearly evident from epidemiological studies in literature [290, 291]. They also promote apoptosis and inhibit angiogenesis by modulating the immune system and structural proteins like sphingolipids and collagen [292, 293]. Moreover, vitamin E stimulates the p53 tumor suppressor gene and simultaneously downregulates mutant p53 proteins [294]. Vitamin E as well as its natural and synthetic isoforms exhibits strong antioxidation potential which further aids in therapies to overcome the side effects of anticancer drugs. Together, these factors prevent cancer development and proliferation [295].

An excellent experimental illustration of the antioxidant protective effect of vitamins and minerals was the famous French study started in 1994, Supplements in Vitamins and Minerals Antioxidants (SU.VI.MAX), a double-blind trial on 5034 men to test the impact of a daily intake of vitamin and mineral antioxidants in reducing the occurrence of prostate cancer. A supplement consisting of a combination of antioxidants at physiological doses of synthetic beta-carotene (6 mg), tocopherol (30 mg), vitamin C (120 mg), selenium (100 *μ*g), and zinc (20 mg) or one placebo capsule was administered daily for 8 years. Overall, there was a moderate, nonsignificant reduction in the incidence of prostate cancer associated with supplementation; however, the effect was significantly different concerning the PSA (prostate-specific antigen). In men with normal initial PSA (<3 ng/mL), the supplement was associated with a significantly reduced rate of prostate cancer, which would support the consumption of antioxidant vitamins and minerals as a method of chemoprevention of prostate cancer [296].

Examining the relationship between MnSOD polymorphisms, a putative risk factor for prostate cancer, and status of exogenous dietary antioxidants—selenium, lycopene, a-tocopherol, and g-tocopherol—in modifying prostate carcinogenesisprocess in 567 cases and 764 controls, Li et al. [297] demonstrated that the combined status of the mentioned antioxidants is inversely associated with the risk of prostate cancer in patients with MnSOD polymorphism.

#### 3.4.3. Polyphenols

Polyphenols comprise more than 8000 hydrosoluble organic compounds, synthesized as secondary metabolites by plants [298, 299]. They are subdivided into several chemical groups and are characterized by the presence of at least two phenolic groups, one of which is directly associated with hydroxyl functions or engaged in another function: ether, ester, and heteroside [300]. Polyphenols are considered powerful antioxidants that prevent oxidative damage by not only trapping hydroxyl radicals, superoxides, and nitrites [301] but also by chelation of transition metals such as iron and copper essential catalysts of redox reactions [302], or by the inhibition of enzymes that produce free radicals [303, 304].

In the EPIC meta-analysis of data obtained from dietary questionnaires of 345,904 people, from seven European countries over a period of six years, Boeing et al. reported a significant inverse association between total fruit and vegetable consumption and risk of cancers of the upper aerodigestive tract of the oral cavity, pharynx, esophagus, and larynx at an 80 g/day intake [305].

Plant flavonoids are considered preventive therapies against cancer [306], and this important role of flavonoids was supported by the results of a recent study of Bondonno et al. analyzing the Danish nationwide dietary behavior over a 23-year period [307]. The researchers noted that a moderate intake of flavonoids was inversely associated with all-cause mortality including the cancer-related mortality, with the strongest associations between flavonoids and mortality being observed in high-risk populations of smokers and high alcohol consumers [307].

Quercetin is a unique flavonoid because of its biphasic oxidation properties. At cell concentrations in the 1-40 mM range, quercetin acts as an antioxidant and thus reduces the ability of ROS to damage cellular DNA [308], whereas at doses above 40 mM, quercetin acts as a prooxidant generating ROS which exert cytotoxic effects on the tumor [308]. Quercetin significantly inhibits NF-*κ*B nuclear factor-kappa B activity activated by ROS [308–311]. The NF-*κ*B is a master regulator of many genes involved in the carcinogenesisprocess through promotion of inflammation, cell growth, differentiation, and angiogenesis [312]. Its activation may regulate the production of prostaglandins via the gene cyclooxygenases-2 (COX-2), which promotes both angiogenesis and metastasis in certain tumor models, potentially through the regulation of vascular endothelial growth factor (VEGF) and MMPs [313–316]. NF-*κ*B mediates a crosstalk between inflammation and the accumulation of proinflammatory cytokines cytokines such as TNF-alpha, and IL-6 in a tumor microenvironment with elevated NF-*κ*B activity leads to the protumorigenic microenvironment [312]. The NF-*κ*B is therefore a promising anticancer therapeutic pathway; the suppression of NF-*κ*B leads to tumor regression [312]. Min and Ebeler revealed that treatments with 1 mM quercetin prevent H_2_O_2_-induced DNA oxidative damage in Caco-2 human epithelial colorectal adenocarcinoma cells by improving the DNA repair process [317]. Moreover, the same researchers observed that treatment with quercetin (1-100 *μ*M) induces the expression of the mRNA of human 8-oxoguanine DNA glycosylase (hOGG1), an enzyme involved in the DNA repair [317]. In addition, quercetin has been shown to inhibit cell proliferation in the human breast carcinoma cell line MCF-7 by inhibiting cell cycle progression at G2 and M phases, in addition to induction of the apoptosis pathway [318]. This mechanism involved was the inhibition of cyclin B1-Cdc2 kinase enzymatic complex activity [318], critically involved otherwise in the unfolding of early mitotic events [319].

## 4. Nanoantioxidant Strategies

In spite of the anticancer effectiveness of antioxidants, they remain a growing challenge for clinicians due to their instability, limited bioavailability, poor solubility, and low selectivity, which limit their therapeutic uses in cancer. To overcome these pharmacokinetic limitations, a promising nanotechnology offers the possibility of delivering bioactive compounds directly into tumor tissues, and therefore providing their maximal anticancer therapeutic activities. A variety of novel nanoantioxidant-based delivery systems to target cancer have been reported [320].

### 4.1. Lipid-Based Nanoparticles

Solid lipid nanoparticles (SLNs) are the novel generation of submicron-sized colloidal nanocarriers ranging from 50 to 1000 nm where the liquid-lipid has been replaced by a solid-lipid such as mono-, di-, or triglycerides, phospholipids, lipid acids, glyceride mixtures or waxes, and cholesterol and surfactants. SLNs offer relevant characteristics such as site-specific targeting, long-term physical stability, large surface area, and low toxicity and therefore have immense antiproliferative potential in cancer therapy by delivering antioxidant drugs incorporating them into the SLN formulation in order to improve the drug pharmacokinetics and/or reduce their toxicities [321]. The SLNs-antioxidant contributions have been reported in curcumin, a natural antioxidant compound with anticancer potential [322]. Wang et al. showed that curcumin- (cur-) loaded SLNs exhibited higher antiproliferative activity in a dose-dependent manner and induced strong cytotoxicity and apoptosis by enhancing ROS production in SKBR3 cells, resulting in a decrease in the Bax/Bcl-2 ratio, as well cyclin-dependent kinase 4 (CDK4) and cyclin D1 expression compared to free curcumin. These findings indicate that cur–SLNs might be an efficient chemodrug against breast cancer [323]. Similar to this, the incorporation of sesame into SLNs showed its ability to induce the normalization of skin cancers after [324].

### 4.2. Micelles

Micelles are defined as the self-assembly of colloidal nanometries (diameter less than 50 nm) and consist of a hydrophobic core and a hydrophilic shell and are therefore used to transport hydrophilic and lipophilic molecules. Their surfactant structures can be aggregated by cationic, anionic, zwitterionic, or nonionic groups depending on the nature of the head groups, their origin, and the length of the alkyl chains. These polymers have been considered promising nanocarriers in cancer clinical trials for their capacity, critical size, drug incorporation efficiency, stability, and release rate [325]. Quercetin-loaded polymeric micelles showed significant antiproliferative and apoptotic potential *in vivo* and *in vitro* in CT26, PC3, and H22 tumor-bearing mice, enhanced cytotoxicity in the MCF-7 human breast cancer cell line, and improved drug accumulation in the A549 cancer cell line and murine xenograft model [326]. Furthermore, Hasegawa et al. demonstrated that administration of catechol in micelles thwart cancer by inhibiting ROS-mediated angiogenesis and thus the formation of capillary networks in the tumor tissue environment in the chicken ex ovo chorioallantoic membrane assay [327].

### 4.3. Cyclodextrin

Cyclodextrins are cyclic oligosaccharides formed from *α*-(1,4)glycosidic units by the enzymatic degradation of starch by cyclodextrin glucanotransferase, resulting in a toroidal structure and are water soluble in nature with a hydrophobic core 0.5 to 1.0 nm in diameter and a hydrophilic outer surface. The cyclodextrin complex confers high aqueous solubility, dissolution rate, and bioavailability to poorly water-soluble drugs. Their unique characteristics predispose them to applications in the design of an efficient cancer drug delivery system [320]. The encapsulation of vitamin E into the inclusion complex *β*-cyclodextrin (*β*-CD) showed fast dissolving nanofibers accompanied with efficient antioxidant properties and light and shelf stability compared to its uncomplexed form. Moreover, even after 3 years of storage, the vitamin E/HP-B-CD nanofibers preserved its antioxidant hallmark. Along the same lines [328], Dhakar et al. reported an improvement of water solubility and antioxidant property with no cytotoxicity of kynurenic acid (4-hydroxyquinoline-2- carboxylic acid, KYNA) loaded in cyclodextrin nanosponges compared to free kynurenic acid [329].

## 5. Antioxidants and Controversies

The antioxidants and ROS display yet controversial or contradictory roles in tumorigenesis, as ROS which more often are involved in tumor development and progression, are also toxic to cancer cells, and can potentially induce apoptosis at high levels [330], and conversely antioxidants may cause an increase in the risk of cancer [331].

Against the general opinion favoring the anticancer role of antioxidants, a 2014 study in mouse models of BRAF and KRAS oncogenes-induced lung cancer found that nutrient supplementation with the antioxidants N-acetylcysteine and vitamin E significantly accelerated tumor cell proliferation and growth by reducing ROS, damaging DNA and preventing tumor suppressor p53 activation [332], which leads to the cessation of its roles in the response to DNA damage such as DNA damage repair, cell cycle arrest, senescence, and apoptosis, all of which are in place to prevent mutations from being passed on down the lineage, its inactivation therefore promotes the development of many types of tumors [332–334].

The above study suggested that the antioxidants in lung cancer cells may downregulate the endogenous ROS defense through feedback mechanism on supplementation with N-acetylcysteine and vitamin E. As a result, a significant decrease can be noted in oxidative DNA damage, which in turn, suppresses the activation of the p53 gene. These outcomes were based on the findings that the antioxidant selectively supported proliferation of lung cancer cells with wild-type p53 and was not observed in cells with mutant or inactivated p53. In general, it is suggested that the continuous use of antioxidants like glutathione, superoxide dismutase, catalase, and thioredoxin may prevent the ROS levels from inducing anticancer mechanisms (mainly apoptosis) by keeping them in check [14]. Also, since tumor cells themselves produce antioxidants to overcome oxidative damage, the additional supplements may allow tumor cells to store surplus antioxidants and promote its survival and further proliferation [335]. A recent observational study of 2014 patients demonstrated an association between antioxidant supplements (including vitamins, carotenoids, and coenzyme Q_10_) and increased risk for breast cancer recurrence and death [336].

In a large-scale research on the potential effects of daily antioxidant supplementation (120 mg of vitamin C, 30 mg of vitamin E, 6 mg of beta-carotene, 100 *μ*g of selenium, and 20 mg of zinc) against the risk of skin cancers, with a median follow-up period of 7.5 years, Hercberg et al. reported more beneficial effects in men, whose prior antioxidant status was lower (for beta-carotene and vitamin C), but not in women, who had an increased rate of melanoma compared to the placebo group [337].

A meta-analysis of four randomized clinical trials involving 109,394 subjects investigating the effect of beta-carotene supplementation on the incidence of lung cancer among smokers and/or male asbestos workers surprisingly showed that high-dose beta-carotene supplementation (30 mg daily) related to tobacco was significantly associated with an increased lung cancer risk; a high concentration of beta-carotene in interaction with cigarette smoke promotes lung carcinogenesis by acting as a prooxidant exacerbating oxidative damage [338]. The reactive nitrogen species (RNS) found in the gaseous phase of cigarette smoke triggers the prooxidant behavior of *β*-carotene, generating wider range of oxidation products, including 4-nitro-*β*-carotene which induced more lipid peroxidation and DNA damage [339–342]. Besides the effect of high concentration, at a high partial pressure of oxygen, may also affect the antioxidants function of *β*-carotene in favor of prooxidant behavior due to autooxidation [343].

## 6. Conclusions

Under physiological conditions, both endogenous and exogenous antioxidants are undoubtedly important and interdependent to circumscribe the prooxidative damage of the ROS. Multiple factors ranging from genetics to imbalanced lifestyle can disrupt oxidative homeostasis leading to oxidative stress, high generation of reactive oxygen species (ROS), and free radicals that consequently assault the structure of DNA molecules, proteins, and increased lipid peroxidation. During these damaging processes, a variety of molecules are produced, including 8-OH deoxyguanosine, malondialdehyde, and 4-hydroxy 2-nonenal, participate to promote the risk of mutagenesis, and are significantly correlated with carcinogenesis.

As such, a strong enzymatic and nonenzymatic antioxidant defense system to efficiently scavenge the ROS and prevent oxidative damage is essential in the prophylaxis against the development of cancerous processes. Numerous reports ranging from *in vitro* and animal experimental to clinical and large observational studies have shown the antitumoral efficacy of the antioxidants and the potential for reducing the side effects of the chemotherapy and radiotherapy regimens. The use of conventional antioxidants-free therapies has been limited in biomedical trials due to their poor permeation across cell membranes and cell internalization, easier degradation, and limited bioavailability. To overcome these barriers, a recent nanoantioxidant system may provide potential solutions to enhance antioxidant efficiency and to offer target delivery.

However, inclusion of antioxidants in cancer therapy is controversial due to their unpredictable interaction with chemodrugs. Furthermore, recent research has revealed the overconsumption of antioxidants (and in particular synthetic antioxidants) can promote the survival and growth of cancer cells; a phenomenon known as the antioxidant paradox. A high antioxidant supplementation that exceeds safe levels can cause “antioxidant stress” and can fuel the metastasis-promoting physiological imbalance. In light of all these data, great caution is advised in the use of large doses of supplements. The natural state of health is a question of balance. A balanced diet of antioxidants can thus hold the key to cancer prevention.

## Figures and Tables

**Table 1:** The role of oxidative stress on cancer development.

Cancer type	Sample collection	Models	Biomarkers	Reference
CRC	Human colon tissue, males, mean age 67 yrs, range 60-83, normal tissue (*n* = 2) and cancerous tissue (*n* = 5)	*In vivo*	↑ROM ↑MPO	Keshavarzian et al. (1992) [87]
	Human colorectal mucosal tissues, normal tissue (*n* = 20) and tissue tumors (*n* = 20)	*In vivo*	↑MDA ↑MPO ↑PLA2	Otamiri et al. (1989) [88]
	Urine sample, cancer patients (*n* = 222) and healthy(*n* = 85) Leukocyte DNA, healthy volunteers (*n* = 134), and malignant cancer patients (*n* = 179)	*In vivo*	↑8-oxoGua ↑8-oxodG ↑8-oxodG	Roszkowski et al. (2011) [91]

BC	Blood samples (plasma), women with breast cancer (*n* = 62) and healthy controls (*n* = 21)	*In vivo*	↑FORT ↓FORD	Tahari et al. (2013) [92]
	Blood samples, women BBC (*n* = 50), BC (*n* = 50), and healthy women (*n* = 50)	*In vivo*	↑8-OHdG	Eldin et al. (2019) [96]
	Urine sample, cancer patients (*n* = 18) or healthy control (*n* = 10)	*In vivo*	↑8-OHdG	Yamamoto et al. (1996) [99]
	Blood samples (serum), BC patients (*n* = 35) and healthy controls (*n* = 35)	*In vivo*	↑MDA ↑GSSG ↓GSH ↓TAC ↓GSH/GSSG	Hewala et al. (2019) [93]

PC	DU 145 LNCaP PC3	*In vitro*	↑H_2_O_2_ ↑O_2_^−^ ↑Nox	Kumar et al. (2008) [107]
	Blood samples (erythrocytes and plasma), PC patients (*n* = 30; age 61 ± 8 years), BPH patients (*n* = 30; age 63 ± 8 years), and healthy men (*n* = 25; age 61 ± 14 years).	*In vivo*	↓GPx3	Szewczyk-Golec et al. (2015) [108]
	Human prostate tissue, PC (*n* = 32), BPH (*n* = 40) patients, and healthy subjects (*n* = 39)	*In vivo*	↓GSH-Px	Zachara et al. (2005) [109]

LC	A/J mice (female *n* = 35)	*In vivo*	↑8-OHdG	Xu et al. (1992) [119]

Abbreviations: 8-OHdG: 8-hydroxy-20–deoxyguanosine; 8-oxodG: 8-oxo-2′-désoxyguanosine; PLA2: phospholipase A2; MP: myeloperoxidase; CRC: colorectal cancer; BC: breast cancer; ROM: reactive oxygen metabolites; GSH: glutathione (total, reduced); GSSG: glutathione disulfide; LC: lung cancer; MDA: malondialdehyde; TAC: total antioxidant capacity; PC: prostate cancer.

## Data Availability

All data used is available on request.
